# YTHDC1 is downregulated by the YY1/HDAC2 complex and controls the sensitivity of ccRCC to sunitinib by targeting the ANXA1-MAPK pathway

**DOI:** 10.1186/s13046-022-02460-9

**Published:** 2022-08-17

**Authors:** Wei Li, Kun Ye, Xurui Li, Xinlin Liu, Mou Peng, Fang Chen, Wei Xiong, Yinhuai Wang, Liang Zhu

**Affiliations:** 1 Department of Urology, The Second Xiangya Hospital, Central South University, 410011 Changsha, Hunan China; 2 Uro-Oncology Institute of Central South University, 410011 Changsha, Hunan China

**Keywords:** Clear cell renal cell carcinoma, YTHDC1, ANXA1, HDAC2/YY1, Sunitinib

## Abstract

**Background:**

Tyrosine kinase inhibitors (TKIs) such as sunitinib are multitarget antiangiogenic agents in clear cell renal cell carcinoma (ccRCC). They are widely used in the treatment of advanced/metastatic renal cancer. However, resistance to TKIs is common in the clinic, particularly after long-term treatment. YTHDC1 is the main nuclear reader protein that binds with m^6^A to regulate the splicing, export and stability of mRNA. However, the specific role and corresponding mechanism of YTHDC1 in renal cancer cells are still unclear.

**Methods:**

The Cancer Genome Atlas (TCGA) dataset was used to study the expression of YTHDC1 in ccRCC. Cell counting kit-8 (CCK-8), wound healing, Transwell and xenograft assays were applied to explore the biological function of YTHDC1 in ccRCC. Western blot, quantitative real time PCR (RT‒qPCR), RNA immunoprecipitation PCR (RIP-qPCR), methylated RIP-qPCR (MeRIP-qPCR) and RNA sequencing (RNA-seq) analyses were applied to study the YY1/HDAC2/YTHDC1/ANXA1 axis in renal cancer cells. The CCK-8 assay and xenograft assay were used to study the role of YTHDC1 in determining the sensitivity of ccRCC to sunitinib.

**Results:**

Our results demonstrated that YTHDC1 is downregulated in ccRCC tissues compared with normal tissues. Low expression of YTHDC1 is associated with a poor prognosis in patients with ccRCC. Subsequently, we showed that YTHDC1 inhibits the progression of renal cancer cells via downregulation of the ANXA1/MAPK pathways. Moreover, we also showed that the YTHDC1/ANXA1 axis modulates the sensitivity of tyrosine kinase inhibitors. We then revealed that HDAC2 inhibitors resensitize ccRCC to tyrosine kinase inhibitors through the YY1/HDAC2 complex. We have identified a novel YY1/HDAC2/YTHDC1/ANXA1 axis modulating the progression and chemosensitivity of ccRCC.

**Conclusion:**

We identified a novel YY1/HDAC2/YTHDC1/ANXA1 axis modulating the progression and chemosensitivity of ccRCC.

**Supplementary Information:**

The online version contains supplementary material available at 10.1186/s13046-022-02460-9.

## Background

Clear cell renal cell carcinoma (ccRCC) is the main type of renal carcinoma [1]. Although surgical treatment is the mainstay of treatment for renal cancer, patients with unresectable advanced renal cancer or recurrent renal cancer rely on systemic treatment, typically antiangiogenic or immunotherapeutic treatment, to prolong their lives [2, 3]. Tyrosine kinase inhibitors (TKIs) such as sunitinib are multitarget antiangiogenic agents in renal carcinoma cells [4]. TKIs are widely used in the treatment of advanced/metastatic renal cancer [4]. However, resistance to TKIs is common in the clinic, particularly after long-term treatment [5]. The mechanism of drug resistance may be related to proangiogenic signaling pathways, changes in the tumor microenvironment, increased tumor invasion and metastasis, microRNA-mediated drug resistance or the activation of other signaling pathways [5]. However, the factors associated with TKI resistance in ccRCC have not been fully clarified. Thus, further study of the mechanism of TKI resistance would be helpful for the development of new antitumor strategies for ccRCC.

N6-Methyladenosine (m^6^A) is the most abundant modification of internal eukaryotic mRNA [6]. m^6^A is associated with mRNA metabolism, folding, processing, export, translation and stability [7]. It has been indicated that m6A modification is closely related to various types of tumors such as lung cancer, acute myeloid leukemia (AML), and hepatocellular carcinoma (HCC) [8]. Renal cancer, particularly clear cell renal cell carcinoma (ccRCC), is correlated with m6A modification [9]. Zhou et al. found that changes in m6A-regulating factors were correlated with pathological stage in ccRCC [9]. There are three groups of molecules that regulate m6A modification: m6A “writers” (METTL14, METTL3, and WTAP), m^6^A “erasers” (ALKBH5 and FTO) and m^6^A “readers” (YTHDC1, HNRNPC, HNRNPA2B, YTHDF1, YTHDF2 and YTHDF3) [10]. m^6^A readers are signal transducers that regulate mRNA modification and are dysregulated in renal cell carcinoma [11–13]. Felix et al. analyzed the expression of six m^6^A readers in ccRCC and found that only the mRNA and protein levels of YTHDC1 were consistently downregulated in ccRCC [13]. Of note, YTHDC1 is the main nuclear reader protein that binds with m^6^A to regulate the splicing, export and stability of mRNA [13–16]. However, the specific role and corresponding mechanism of YTHDC1 in renal cancer cells are still unclear.

Consistent with previous findings, we demonstrated that YTHDC1 is downregulated in ccRCC and that low expression of YTHDC1 is correlated with a poor prognosis. We then showed that YTHDC1 participates in inhibiting the progression of ccRCC. Interestingly, we found that YTHDC1 represses the expression of ANXA1, which was previously reported to modulate TKI resistance in ccRCC [17]. Moreover, we revealed that the HDAC2/YY1 complex regulates the expression of YTHDC1 in ccRCC and that HDAC2 inhibitors could sensitize ccRCC cells to TKI treatment through YTHDC1. Thus, we identified a novel HDAC2/YY1/YTHDC1/ANXA1 axis in ccRCC.

## Materials and methods

### Cell lines and cell culture

The renal cell carcinoma cell lines 786-O (#CL-0010, Procell Life Science & Technology, Wuhan, China) and A498 (#CL-0254, Procell Life Science & Technology, Wuhan, China) were identified by short tandem repeat (STR) profiling using Procell Life Science & Technology. The 786-O cells were cultured in RPMI-1640 medium (Gibco, USA), and the A498 cells were cultured in MEM (PM150410, Procell Life Science & Technology) supplemented with 10% fetal bovine serum (FBS) (AC03L055, Shanghai Life-iLab Biotech, China) and incubated at 37 °C in 5% CO_2_. Sunitinib-resistant 786-O cells (786-O R) were generated as previously reported [17].

The chemicals used were as follows: The HDAC2 inhibitors included santacruzamate A (CAY10683) (# S7595, Selleck, China), LY3214996 (#S8534, Selleck, China), GSK1120212 (#S2673, Selleck, China), and actinomycin D (#S8964, Selleck, China). The siRNA was obtained from RiboBio (Guangzhou, China). The shRNAs were purchased from GeneCopoeia (USA). The sequences of the siRNA and shRNA are provided in Table S1.

### Western blot analysis and antibodies

The western blot analysis procedure was reported previously. In brief, the cells were lysed and the proteins extracted with RIPA buffer (#P0013, Beyotime, China). The protein content was quantified by the BCA method. Proteins were boiled and subjected to sodium dodecyl sulfate‒polyacrylamide gel electrophoresis (SDS‒PAGE) separation. The proteins were transferred onto 0.45 μm polyvinylidene fluoride membranes (Millipore, USA) and incubated with the corresponding primary antibodies and secondary antibodies. Protein signals were visualized using ECL detection reagent (Thermo Fisher Scientific, USA) and ChemiDoc XRS (Bio-Rad Laboratories, USA). The primary antibodies used were as follows: YTHDC1 (#29441-1-AP, Proteintech, 1:500 dilution), ANXA1 (#55018-1-AP, Proteintech, 1:2000 dilution), YY1 (#66281-1-Ig, Proteintech, 1:1000 dilution), HDAC2 (#12922-3-AP, Proteintech, 1:1000 dilution), and beta actin (#66009-1-Ig, Proteintech, 1:5000 dilution).

### Immunohistochemistry (IHC)

Immunohistochemistry was performed with primary antibodies against YTHDC1 (#29441-1-AP, Proteintech, 1:1000, ANXA1 (21990-1-AP, Proteintech; 1:10000 dilution) and TMA slides (#KD1921, Avilabio, China). The extent of binding was evaluated based on the proportion of positively stained tumor cells and the staining intensity score. The staining intensity score was as follows: 0 (no staining), 1 (weak staining = light yellow), 2 (moderate staining = yellow brown), and 3 (strong staining = brown). A total score was obtained by multiplying the intensity score by the proportion of cells stained.

### Quantitative real-time PCR (RT‒qPCR) and chromatin immunoprecipitation (ChIP)-qPCR analysis

The details of RT‒qPCR were described previously [17]. In brief, TRIzol reagent (Thermo Fisher Scientific, USA) was used to extract total RNA from cells. A reverse transcription kit and PCR kit (#RR037A PrimeScript™ RT reagent Kit and #RR430A TB Green™ Fast qPCR Mix, respectively, Takara Bio Inc. Shigo, Japan) were used to perform the RT‒qPCR assay. The primer sequences for RT‒PCR are provided in Table S2. A chromatin extraction kit (Abcam, ab117152, USA) and the ChIP Kit Magnetic-One Step (Abcam, ab156907, USA) were used to perform ChIP‒qPCR. The primer sequences for ChIP‒PCR are provided in Table S3.

### Cell counting Kit-8 (CCK-8) assay

The 786° or A498 cells were seeded in 96-well plates; approximately 10^4^ cells were seeded per well. After culturing for 24 h at 37 °C in 5% CO_2_, the cells were divided into several groups with different treatments; each group had at least 3 repetitions. Ten microliters of CCK-8 reagent (#C0037, Beyotime, China) was added to each well and incubated for 1 h under the above conditions. The absorbance at 450 nm was measured by a microplate reader.

### Nude mouse xenograft assay

Because the gender of the mice had no effect on the results of the study, we chose half males and half females for the experiment. BALB/C-nu/nu mice (Hunan SJA Laboratory Animal Company), 6 weeks old and 22–24 g, were individually housed in the animal center of the Second Xiangya Hospital under 60 ± 3% humidity at 22 ± 0.5 °C; the light/dark cycle was controlled automatically. Animals had free access to food and water. The renal carcinoma cells were injected subcutaneously on the left side of the back of the mouse (1 × 10^7^ cells per mouse). The length and width of the tumor were measured with a Vernier caliper every 2 d, and the tumor volume was calculated using the formula (L × W^2^)/2. Mice were sacrificed at the appropriate time, and the tumors were collected for further study. All animal experiments were reviewed and approved by the Institutional Animal Care and Use Committee (IACUC) of the Second Xiangya Hospital, Central South University (animal license number 2,021,095). The animal experiment complied with the National Institutes of Health Guide for the Care and Use of Laboratory Animals (NIH Publications No. 8023, revised 1978). Because a minimum of four mice per group is necessary to compare differences between two groups, in the nude mouse xenograft assays, six mice per group were originally established for both the 786-O and A498 cell lines. However, in the A498 group, two mice died unnaturally during the course of the experiment. Thus, in the in vivo experimental design, the 786-O cell line comparison was 6 vs. 6, and the A498 cell line comparison was 4 vs. 4.

### RNA immunoprecipitation (RIP) and Methylated (Me) RIP-qPCR

Whole cells were lysed with 1 × RIPA buffer (Beyotime Biotech, China), and 10% of the lysates were collected as input. The primary antibody or IgG and protein A + G beads (#P2029, Beyotime, China) were then added to the rest of the cell lysate and incubated on a shaker at 4 ℃ for 12 h. The beads were washed with 1 × RIPA buffer, resurrected with protein kinase K buffer and incubated with shaken RNAiso Plus (TaKaRa, 9109) at 55 ℃ for 30 min. RT‒qPCR was performed using the reverse transcription kit and the polymerase chain reaction kit according to the manufacturer’s instructions. The primer sequences are provided in Table S4. The Magna M6A MeRIP kit (# A-17-10499, A&D Technology, Beijing, China) was used to perform MeRIP-qPCR according to the manufacturer’s protocols. The primer sequences are provided in Table S5.

### Statistical analysis

The experimental data came from three independent experiments and are presented as the mean ± standard error of the mean (mean ± SEM). GraphPad Prism 5 software was used to calculate the *P* value using an unpaired two-sided Student’s t test to compare values between two groups or a one-way analysis of variance (ANOVA) followed by Tukey’s multiple comparisons post hoc test to compare values between more than two groups. The sample size (n) for each statistical analysis is provided in the figure legends. Differences were considered statistically significant when the *P* values were less than 0.05. In all cases, the significance of differences was indicated as follows: *P < 0.05; **P < 0.01; ***P < 0.001; and not significant (ns), *P* > 0.05.

## Results

### Downregulation of YTHDC1 promotes the progression of ccRCC *in vivo* and *in vitro*

**Fig. 1:**
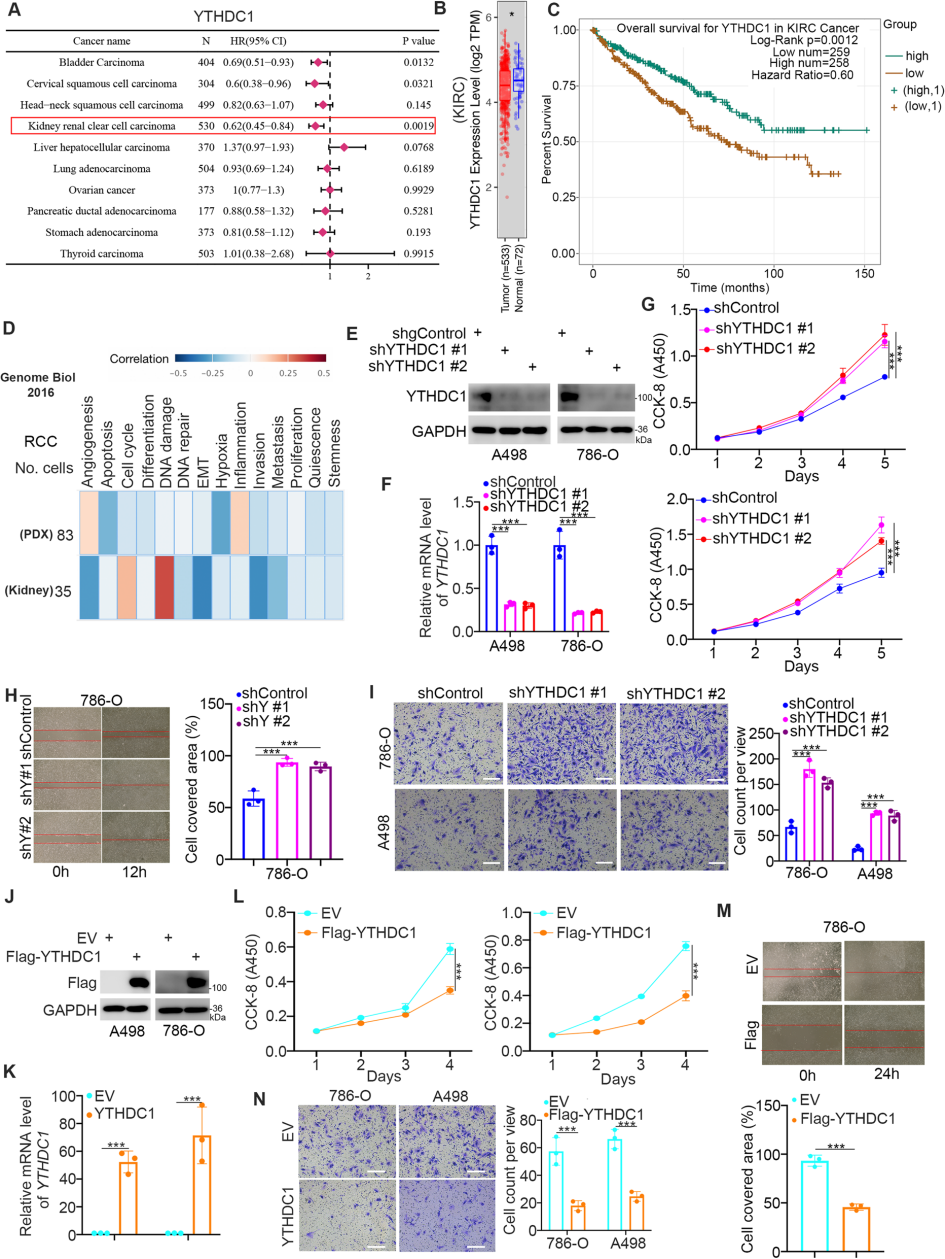
The downregulation of YTHDC1 is associated with the poor prognosis in ccRCC. **A**, The expression level of YTHDC1 was analyzed by the ENCORI web tool in various types of solid tumor. P values and HR were indicated in the panel. **B**, The expression level of YTHDC1 in KIRC was analyzed by the Timer 2.0 (http://timer.cistrome.org/). *, *P* < 0.05. **C**, The prognosis of YTHDC1 in KIRC was determined by the ENCORI web tool. P values as indicated. **D**, CancerSEA web tool was used to analyze the biological function of YTHDC1 in the renal cell carcinoma. **E-I**, A498 and 786-O cells were transfected with indicated shRNAs for 72 h. After puromycin selection, cells were harvested for western blot analysis (**E**), RT-qPCR assay (**F**), CCK-8 assay (**G**), wound healing assay (**H**), transwell assay (**I**). Data presents as mean ± SD with three replicates. ***, *P* < 0.001. **J-N**, A498 and 786-O cells were transfected with indicated plasmids for 24 h. Cells were harvested for western blot analysis (**J**), RT-qPCR assay (**K**), CCK-8 assay (**L**), wound healing assay (**M**), transwell assay (**N**). Data presents as mean ± SD with three replicates. ***, *P* < 0.001

**Fig. 2:**
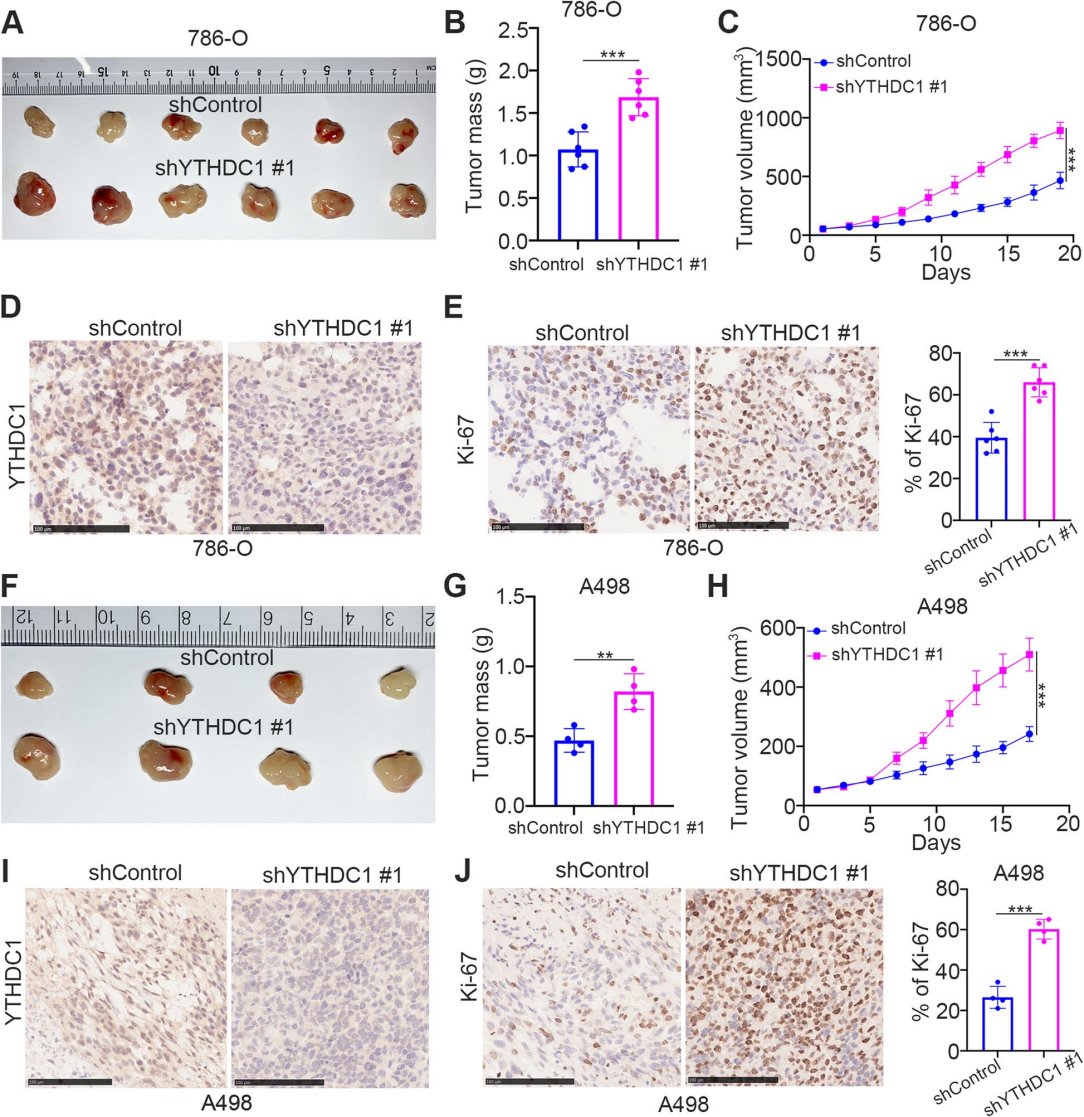
Knockdown of YTHDC1 promotes the progression of ccRCC *in vivo*. **A-E**, 786-O cells were transfected with indicated shRNAs for 72 h. After puromycin selection, cells were subcutaneously injected into the nude mice. The tumor image was shown in panel **A**, the tumor mass was shown in panel **B**, the tumor growth curve was shown in panel **C**. The excised tumors were subjected to IHC staining of YTHDC1 (**D**) or Ki-67 (**E**). Data presents as mean ± SD with six replicates. ***, *P* < 0.001. **F-J**, A498 cells were transfected with indicated shRNAs for 72 h. After puromycin selection, cells were subcutaneously injected into the nude mice. The tumor image was shown in panel F, the tumor mass was shown in panel **G**, the tumor growth curve was shown in panel **H**. The excised tumors were subjected to IHC staining of YTHDC1 (**I**) or Ki-67 (**J**). Data presents as mean ± SD with four replicates. **, *P* < 0.01; ***, *P* < 0.001

We first studied the clinical characteristics of YTHDC1 in malignant tumors. Interestingly, we showed that YTHDC1 was profoundly downregulated in ccRCC with a hazard ratio (HR) of less than 1.0 and *P* values less than 0.05 by analyzing the ENCORI dataset (https://starbase.sysu.edu.cn/panCancer.php) and the Timer 2.0 dataset (http://timer.cistrome.org/) (Fig. 1A and B). Analysis of the TCGA dataset also demonstrated that low YTHDC1 expression was associated with a poor prognosis in patients with ccRCC (Fig. 1C). These data suggest that YTHDC1 is a potential prognostic predictor in ccRCC. The CancerSEA dataset (http://biocc.hrbmu.edu.cn/CancerSEA/goSearch) was then assessed to predict the cancer-associated role of YTHDC1 in renal cancer cells. We found that YTHDC1 expression was negatively correlated with the proliferation, invasion and metastasis of renal cell carcinoma cells (Fig. 1D). To verify this, YTHDC1 was knocked down by transfection with two independent shRNAs in A498 and 786-O cells (Fig. 1E, F). We showed that YTHDC1 inhibition promoted renal cancer cell proliferation, migration, and invasion in vitro (Fig. 1G and I). Conversely, ectopic overexpression of YTHDC1 impeded the proliferation, migration, and invasion of both A498 and 786-O cells (Fig. 1J-N). In addition, the nude mouse xenograft assay also demonstrated that knockdown of YTHDC1 increased the tumor growth of renal cancer cells (Fig. 2A-J). Thus, our data suggest that YTHDC1 is abnormally downregulated in renal cancer tissues, downregulated YTHDC1 is associated with unfavorable prognosis, and YTHDC1 inhibits the progression of renal cancer in cells and in mice.

### YTHDC1 contributes to the inactivation of the ERK/MAPK signaling pathway in ccRCC cells

**Fig. 3:**
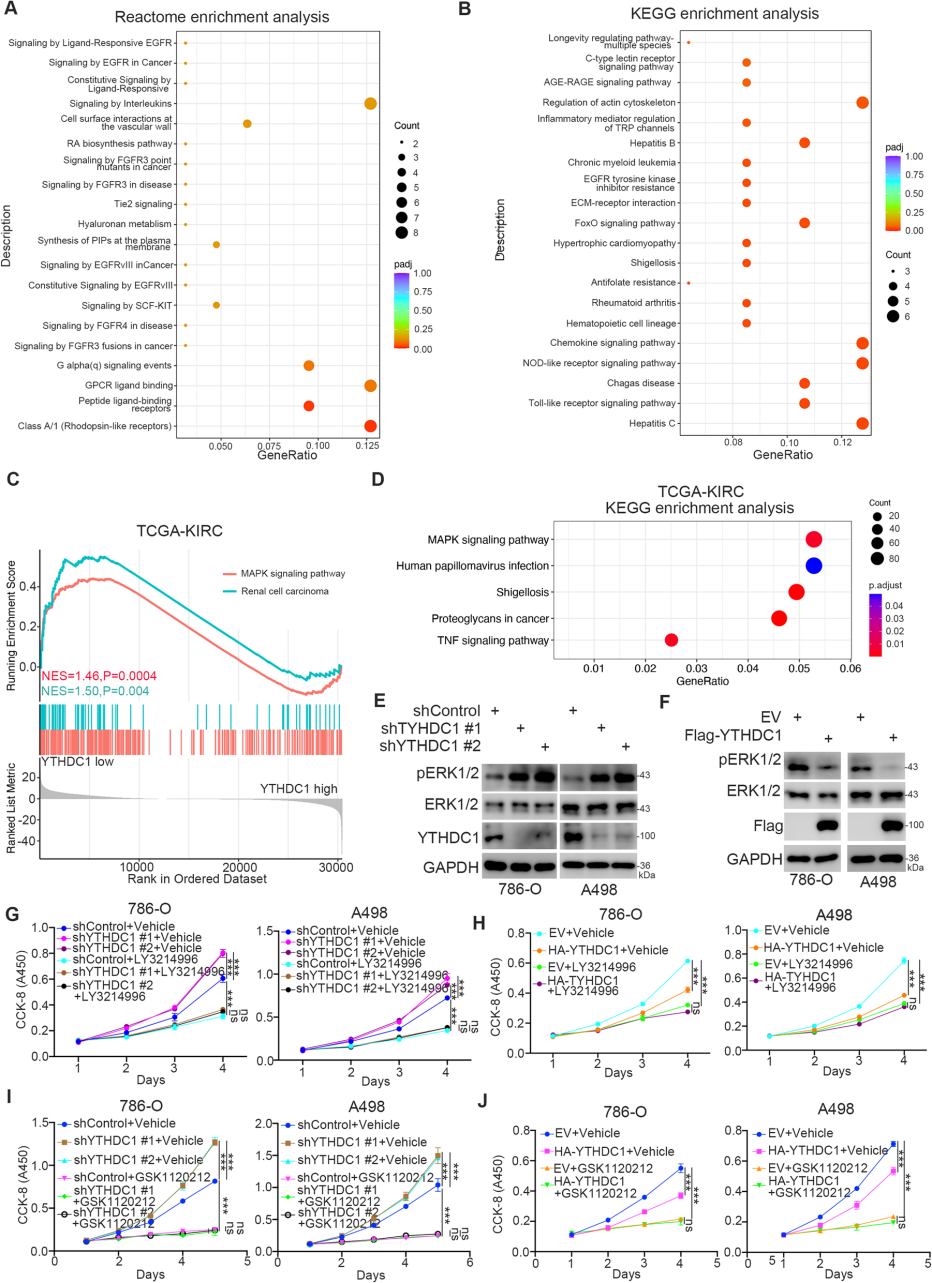
YTHDC1 contributes to the inactivation of the ERK/MAPK signaling pathway in ccRCC cells. **A **and** B**, the Reactome enrichment analysis (**A**) and KEGG enrichment analysis (**B**) of the RNA-seq of YTHDC1 in A498 cells. *P* values as indicated. **C **and **D**, GSEA analysis and KEGG enrichment analysis of the TCGA-KIRC dataset. **E**, 786-O and A498 cells were transfected with the indicates shRNAs for 72 h. After puromycin selection, cells were harvested for western blot analysis. **F**, 786-O and A498 cells were transfected with the indicated plasmids for 24 h. Cells were harvested for western blot analysis. **G**, 786-O and A498 cells were transfected with the indicates shRNAs for 72 h. Then, cells were treated with or without LY3214996 (2 µM) and subjected to CCK-8 assay. **H**, 86-O and A498 cells were transfected with the indicates plasmids for 24 h. Then, cells were treated with or without LY3214996 (2 µM) and subjected to CCK-8 assay. **I**, 786-O and A498 cells were transfected with the indicates shRNAs for 72 h. Then, cells were treated with or without GSK1120212 (20 nM) and subjected to CCK-8 assay. **J**, 86-O and A498 cells were transfected with the indicates plasmids for 24 h. Then, cells were treated with or without GSK1120212 (20 nM) and subjected to CCK-8 assay

Next, we explored the underlying mechanism by which YTHDC1 inhibits the progression of renal cancer cells. Transcriptome sequencing was performed in A498 cells after YTHDC1 knockdown (Supplementary Fig. 1A). Reactome enrichment analysis demonstrated that YTHDC1 was negatively correlated with EGFR and FGFR signaling pathways in renal cancer cells, which are reported to be the major driver pathways for the tumorigenesis of renal cancer [18–20] (Fig. 3A). Of note, a Kyoto Encyclopedia of Genes and Genomes (KEGG) enrichment analysis indicated that YTHDC1 inhibition activated the EGFR tyrosine kinase inhibitor resistance pathway in cancer (Fig. 3B). A Gene Set Enrichment Analysis (GSEA) of the TCGA-KIRC dataset demonstrated that low YTHDC1 expression was positively correlated with renal cell carcinoma and the mitogen-activated protein kinase (MAPK) signaling pathway (Fig. 3C). KEGG enrichment analysis indicated that YTHDC1 inhibition resulted in the activation of the MAPK signaling pathway and the tumor necrosis factor (TNF) signaling pathway in cells (Fig. 3D). It is well known that the extracellular signal-regulated kinases (ERK)/MAPK signaling pathway is responsible for the progression of clear cell renal cell carcinoma [21, 22]. Thus, we sought to determine whether YTHDC1 affects the activation of the MAPK signaling pathway in renal cancer cells. Notably, knockdown of YTHDC1 increased the phosphorylation of ERK, which is a marker of activation of the MAPK signaling pathway, in 786-O and A498 cells (Fig. 3E). In contrast, overexpression of YTHDC1 decreased the phosphorylation of ERK in renal cancer cells (Fig. 3 F). We then demonstrated that treatment with ERK or MEK inhibitors (LY3214996 or GSK1120212, respectively) attenuated the effect of YTHDC1 on suppressing renal cancer cell proliferation (Fig. 3G J). Together, these data indicate that YTHDC1 inactivates the MAPK signaling pathway to inhibit the proliferation of renal cancer cells.

#### YTHDC1 represses ANXA1 expression in ccRCC cells

**Fig. 4:**
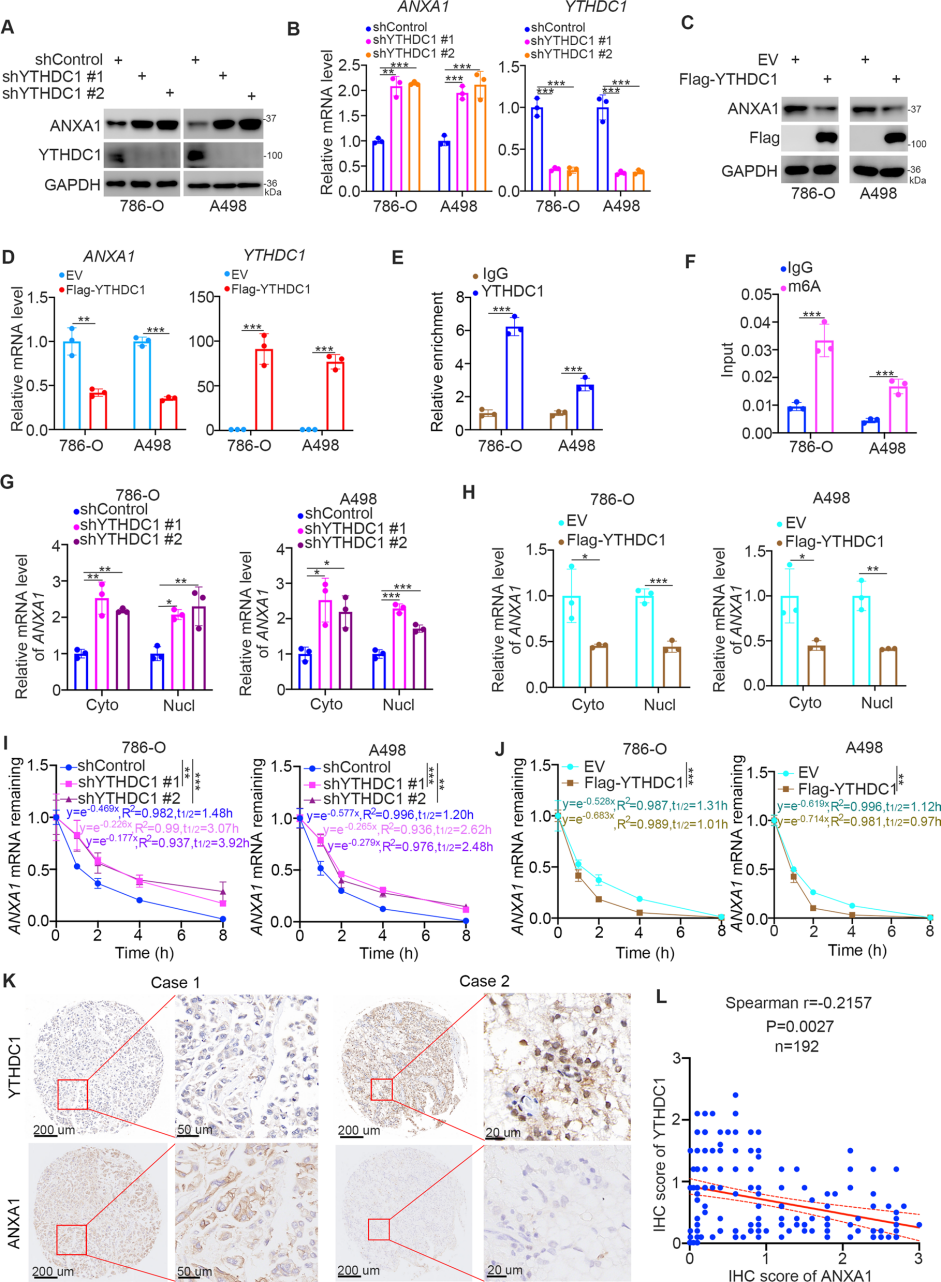
YTHDC1 represses ANXA1 expression in ccRCC cells. **A **and** B**, 786-O and A498 cells were transfected with the indicates shRANs for 72 h. Cell were harvested for western blot analysis and RT-qPCR analysis. Data presents as mean ± SD with four replicates. **, *P* < 0.01; ***, *P* < 0.001. **C **and** D**, 786-O and A498 cells were transfected with the indicates plasmids for 24 h. Cell were harvested for western blot analysis and RT-qPCR analysis. Data presents as mean ± SD with four replicates. **, *P* < 0.01; ***, *P* < 0.001. **E**, The IgG or YTHDC1 antibodies was used to performed the ChIP-qPCR assay in 786-O and A498 cells. Data presents as mean ± SD with four replicates. ***, *P* < 0.001. **F**, Magna m^6^A MeRIP kit was used to performed MeRIP-qPCR assay in 786-O and A498 cells. Data presents as mean ± SD with four replicates. **, *P* < 0.001. **G**, 786-O and A498 cells were transfected with indicated shRNAs for 72 h. Cells were harvested, and the RNA was extracted from the cytoplasm (cyto) or nucleus (mucl) respectively. The RT-qPCR assay was used to detect the mRNA of ANXA1 in 786-O and A498 cell. Data presents as mean ± SD with four replicates. *, *P* < 0.05; **, *P* < 0.01; ***, *P* < 0.001. **H**, 786-O and A498 cells were transfected with indicated plasmids for 24 h. Cells were harvested, and the RNA was extracted from the cytoplasm (cyto) or nucleus (mucl) respectively. The RT-qPCR assay was used to detect the mRNA of ANXA1 in 786-O and A498 cell. Data presents as mean ± SD with four replicates. *, *P* < 0.05; **, *P* < 0.01; ***, *P* < 0.001. **I**, 786-O and A498 cells were transfected with indicated shRNAs for 72 h. Then, cells treated with actinomycin D (5 µg/mL). Then, cells were collected at the different time points. Total RNAs were extracted and analyzed by RT-qPCR. The mRNA expression for each group was normalized to β-actin. **J**, 786-O and A498 cells were transfected with indicated plasmids for 72 h. Then, cells treated with actinomycin D (5 µg/mL). Then, cells were collected at the different time points. Total RNAs were extracted and analyzed by RT-qPCR. The mRNA expression for each group was normalized to β-actin. **K **and** L**, The IHC staining was performed in the tissue microarray of renal cancer by using the YTHDC1 and ANXA1 antibodies. The typical image was shown in panel **K**. The correlation between ANXA1 and YTHDC1 was shown in panel **L**, *P* = 0.0027

As an m^6^A reader, YTHDC1 has been found to regulate mRNA splicing [23], nuclear export [15], and stability [24]. We rechecked the RNA-seq data of YTHDC1 and found that Annexin-A1 (ANXA1) was upregulated after knockdown of YTHDC1 in cancer cells (Supplementary Fig. 1 A and B). Although ANXA1 was not associated with prognosis in patients with renal clear cell carcinoma (Supplementary Fig. 1 C and D), ANXA1 is abnormally upregulated in renal cancer specimens, and inhibition of ANXA1 decreases the proliferation and invasion of renal cancer cells [25]. Interestingly, ANXA1 has been suggested to regulate EGFR activity in malignancy [26]. Moreover, ANXA1 contributes to the ERK-NFκB activation loop in breast cancer cells [27]. Because YTHDC1 has been reported to regulate the expression level of target genes by influencing their mRNA stability [24], we speculated whether ANXA1 was the downstream gene of YTHDC1 in renal cancer cells. First, knockdown of YTHDC1 increased the protein and mRNA levels of ANXA1 in 786-O and A498 cells (Fig. 4A and B). Meanwhile, we also showed that YTHDC1 inhibition led to the up-regulation of ANXA1 in xenografts derived from the 786-O cells (Supplementary Fig. 2 A). In contrast, overexpression of YTHDC1 decreased ANXA1 expression in renal cancer cells (Fig. 4C and D). We then applied the Encyclopedia of RNA Interactomes (ENCORI) dataset to explore the stacked peak regions of YTHDC1 on ANXA1. There were two stacked regions (chr9, 75,773,656–75,773,682; chr9, 75,778,393–75,778,412) on ANXA1 (Supplementary Fig. 1E). We assessed the m^6^A modification sites of ANXA1 using the RMBase v2.0 (https://rna.sysu.edu.cn/rmbase/index.php) web tool (Supplementary Fig. 1F), and we found that the chr9 75,778,393–75,778,412 region is simultaneously bound by YTHDC1 and tagged with an m^6^A modification (Supplementary Fig. 1E and F). A RIP assay using YTHDC1 antibodies was performed around the chr9 75,778,393–75,778,412 region. We showed that YTHDC1 binds to ANXA1 in 786-O and A498 cells (Fig. 4E). Moreover, m^6^A modification was also observed in this region (Fig. 4F). Given that YTHDC1 is also responsible for RNA translocation [28], we knocked down or overexpressed YTHDC1 but found no changes in the subcellular localization of ANXA1 in either 786-O or A498 cells (Fig. 4G H). Interestingly, we showed that YTHDC1 inhibition increased the stability of ANXA1 mRNA, but overexpression of YTHDC1 decreased the stability of ANXA1 mRNA in 786-O and A498 cells (Fig. 4I J). Moreover, IHC staining of the tissue microarray indicated that YTHDC1 was negatively correlated with ANXA1 in renal cancer (Fig. 4K and L). Furthermore, we stained the nude mouse xenograft sample with ANXA1 and YTHDC1 antibodies. We showed that ANXA1 was upregulated after YTHDC1 knockdown (Supplementary Fig. 2). Therefore, these results suggest that YTHDC1 decreases the mRNA stability of ANXA1 in renal cancer cells.

#### YTHDC1 inhibits activation of the MAPK signaling pathway by targeting ANXA1 in ccRCC

**Fig. 5:**
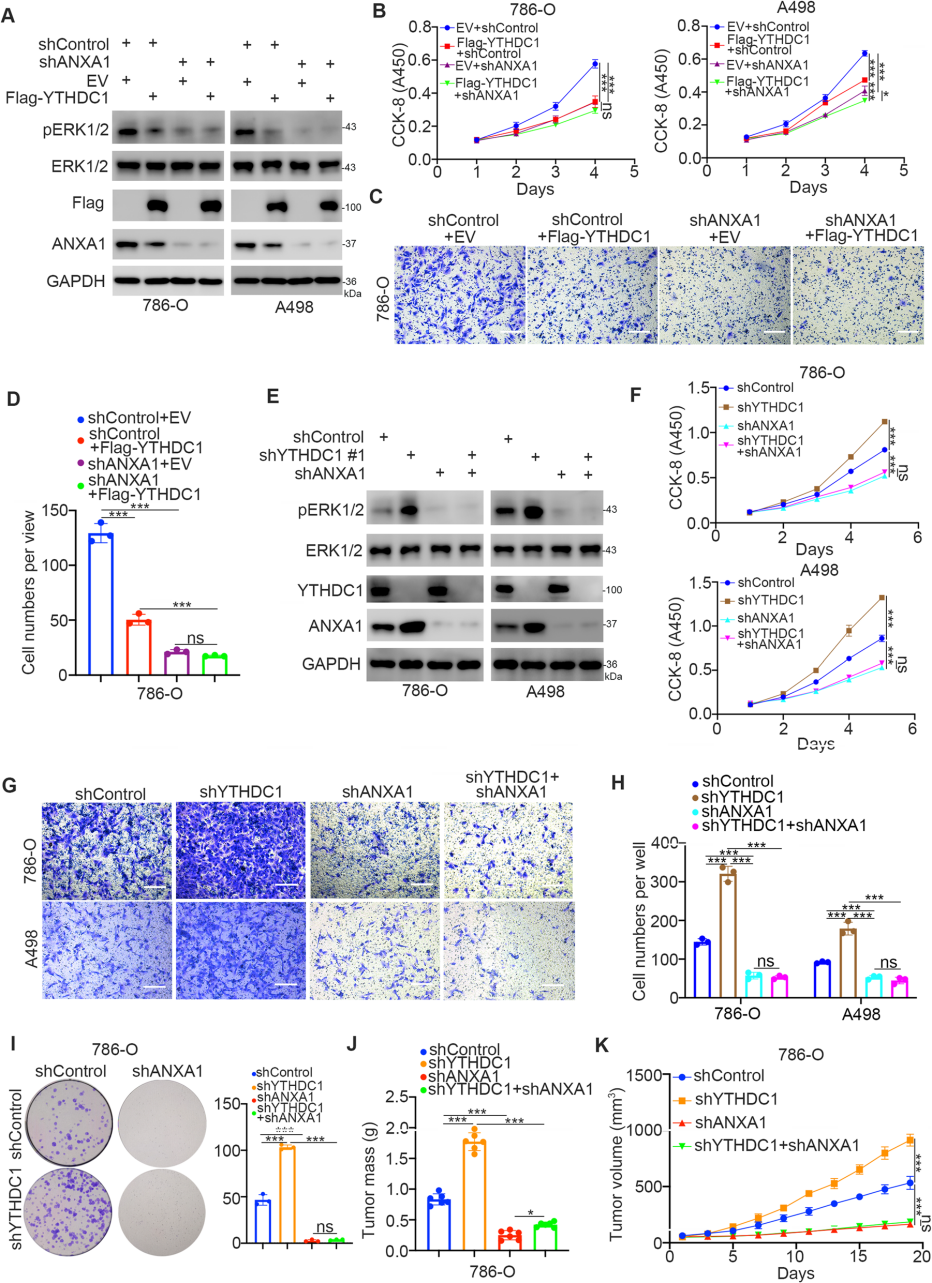
YTHDC1 inhibits the activation of the MAPK signaling pathway by targeting ANXA1 in ccRCC. **A-D**, The 786-O and A498 cells were transfected with indicated constructs for 48 h. Cells were harvested for western blot analysis (**A**), CCK-8 assay (**B**), transwell assay (**C** and **D**). Data presents as mean ± SD with three replicates. Ns, not significant; *, *P* < 0.05; ***, *P* < 0.001. **E-I**, The 786-O and A498 cells were transfected with indicated shRNAs for 72 h. After puromycin selection, cells were harvested for western blot analysis (**E**), CCK-8 assay (**F**), tranwell assay (**G** and **H**) and colonformation assay. Data presents as mean ± SD with three replicates. Ns, not significant; *, *P* < 0.05; ***, *P* < 0.001. **J **and** K**, 786-O cells were transfected with indicated shRNAs for 72 h. After puromycin selection, cells were collected and subcutaneously injected into the nude mice. The tumor mass and tumor growth curve were shown in panel **J** and panel **K**. Data presents as mean ± SD with six replicates. Ns, not significant; *, *P* < 0.05; ***, *P* < 0.001

**Fig. 6:**
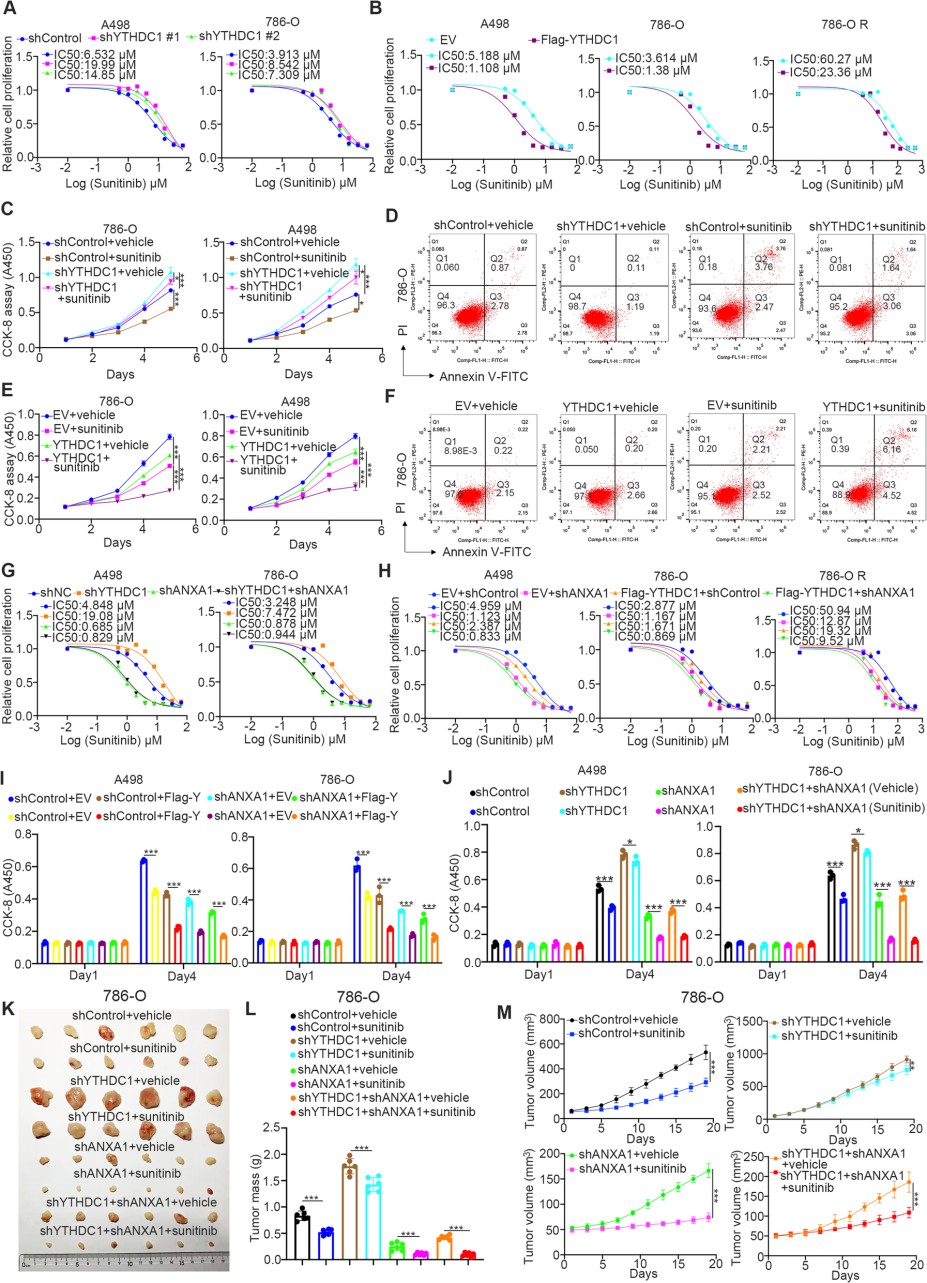
The YTHDC1-ANAX1 axis regulates the sensitivity of sunitinib in ccRCC. **A**, A498 and 786-O cells were transfected with indicated shRNAs for 72 h. Cells were treated with a serial dose of sunitinib for 24 h and harvested for CCK-8 assay. **B**, A498, 786-O and 786-O R (sunitinib resistance) cells were transfected with indicated plasmids for 72 h. Cells were treated with a serial dose of sunitinib for 24 h. Cells were harvested for CCK-8 assay. **C **and** D**, 786-O and A498 cells were transfected with indicated shRNAs for 72 h. Cells were treated with or without sunitinib (2 µM) and subjected to CCK-8 assay (**C**) and Annexin V-PI assay (**D**). Data presents as mean ± SD with three replicates. *, *P* < 0.05; ***, *P* < 0.001. **E **and** F**, 786-O and A498 cells were transfected with indicated plasmids for 24 h. Cells were treated with or without sunitinib (2 µM) and subjected to CCK-8 assay (**E**) and Annexin V-PI assay (**F**). Data presents as mean ± SD with three replicates. **, *P* < 0.01; ***, *P* < 0.001. **G**, A498 and 786-O cells were transfected with indicated shRNAs for 72 h. Cells were treated with a serial dose of sunitinib for 24 h and harvested for CCK-8 assay. **H**, A498 and 786-O cells were transfected with indicated constructs for 48 h. Cells were treated with a serial dose of sunitinib for 24 h and harvested for CCK-8 assay. **I**, A498 and 786-O cells were transfected with indicated constructs for 48 h. Cells were harvested for CCK-8 assay. Data presents as mean ± SD with three replicates. ***, *P* < 0.001. **J**, A498 and 786-O cells were transfected with indicated constructs for 72 h. Cells were harvested for CCK-8 assay. Data presents as mean ± SD with three replicates. *, *P* < 0.05; ***, *P* < 0.001. **K-M**, 786-O cells were transfected with indicated shRNAs for 72 h. After puromycin selection, cells were collected and subcutaneously injected into the nude mice. These mice were treated with or without sunitinib. The tumor mass and tumor growth curve were shown in panel **L** and panel **M**. Data presents as mean ± SD with six replicates. **, *P* < 0.05; ***, *P* < 0.001

Because ANXA1 controls MAPK activation in cells [29, 30], we investigated whether YTHDC1 inhibits the MAPK pathway through ANXA1. We constructed renal cancer cells with stable ANXA1 knockdown induced by transfection with ANXA1-specific shRNAs following puromycin selection (Fig. 5A). YTHDC1 was then overexpressed in shControl or shANXA1 cells by transfection with Flag-tagged YTHDC1 plasmids (Fig. 5A). We found that overexpression of YTHDC1 decreased the phosphorylation of ERK in the shControl group but had no obvious effect on the phosphorylation of ERK in the shANXA1 group in renal cancer cells (Fig. 5A). Similarly, we demonstrated that the inhibitory effect on cell proliferation or invasion after overexpression of YTHDC1 was diminished in shANXA1 renal cancer cells (Fig. 5B and D). In contrast, we constructed ANXA1, YTHDC1 knockdown alone, or ANXA1 + YTHDC1 coknockdown renal cancer cells (Fig. 5E). We found that ANXA1 repression could also attenuate the effect of YTHDC1 knockdown on the change in ERK phosphorylation. Furthermore, the CCK-8 assay, colony formation assay, nude mouse xenograft assay (the tumor image is indicated in Fig. 6K) and Transwell assay showed that the change in cell proliferation ability and invasion capability after knockdown of YTHDC1 was diminished after coknockdown of ANXA1 in 786-O and A498 cells (Fig. 5F-K). Thus, our data indicate that the YTHDC1-ANXA1 axis contributes to the activation of the MAPK signaling pathway in renal cancer cells.

#### The YTHDC1-ANXA1 axis regulates sunitinib sensitivity in ccRCC

Tyrosine kinase inhibitors such as sunitinib are the first-line treatment for advanced renal cell carcinoma [31, 32]. Emerging resistance to sunitinib has become the major obstacle for prolonging the survival of patients with renal cell carcinoma [33, 34]. The KEGG enrichment analysis of the RNA-seq of YTHDC1 showed that YTHDC1 was involved in the EGFR tyrosine kinase inhibitor resistance pathways in A498 cells (Fig. 3B). We previously reported that ANXA1 participates in modulating the sensitivity to sunitinib in ccRCC [17]. The literature has also documented that the aberrant activation of MEK-ERK signaling induces sunitinib resistance in renal cancer [35, 36]. It is not surprising that YTHDC1 knockdown increased the IC50 values of sunitinib in A498 and 786-O cells (Fig. 6A). Ectopic overexpression of YTHDC1 reduced the IC50 values of sunitinib in A498, 786-O and sunitinib-resistant 786-O (786-O R) cells, as reported previously [17] (Fig. 6B). The CCK-8 assay and Annexin V-FITC/propidium iodide staining assay demonstrated that knockdown of YTHDC1 decreased the sensitivity of renal cancer cells to sunitinib (Fig. 6C and D, Supplementary Fig. 3 A), but overexpression of YTHDC1 led to sunitinib resistance in renal cancer cells (Fig. 6E F, Supplementary Fig. 3B). Moreover, we showed that ANXA1 depletion could attenuate the change in the IC50 values of sunitinib induced by knockdown or overexpression of YTHDC1 in both A498 and 786-O cells (Fig. 6G H). Subsequent in vitro and in vivo cell proliferation studies indicated that knockdown of ANXA1 blocked the effect of YTHDC1 on modulating the sensitivity of renal cancer cells to sunitinib (Fig. 6I M). Together, our results demonstrate that YTHDC1 regulates the sensitivity of renal cancer cells to sunitinib through ANXA1.

#### The YY1/HDAC2 complex downregulates the expression of YTHDC1 in ccRCC

**Fig. 7:**
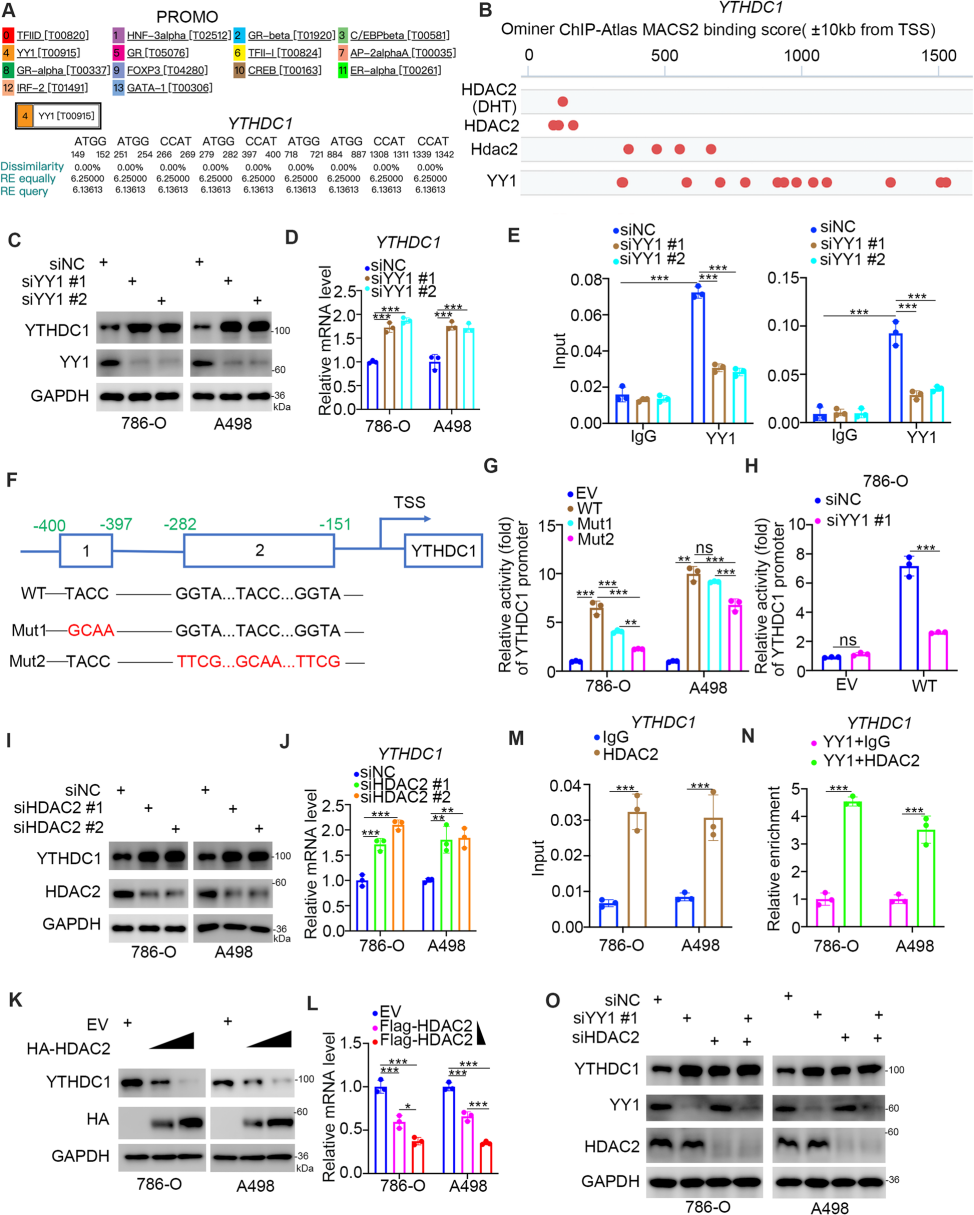
The YY1/HDAC2 complex downregulates the expression of YTHDC1 in ccRCC. **A**, The PROMO web tool predicted the potential transcriptional factors of YTHDC1. **B**, The Ominer web tool predicted the potential transcriptional factors of YTHDC1. **C-E**, 786-O and A498 cells were transfected with indicated siRNAs for 48 h. Cells were collected for western blot analysis (**C**), RT-qPCR assay (**D**), and ChIP-qPCR assay (**E**). Data presents as mean ± SD with three replicates. ***, *P* < 0.001. **F**, a diagram demonstrated the sequence and position of the YY1 binding peak in the YTHDC1 promoter. TSS transcriptional start site, WT wild type, MUT mutant type. **G**, 786-O and A498 cells were transfected with empty vector, GV592-YTHDC1 plasmids WT, MUT1, or MUT2 for 48 h. Cells were harvested and the activity of YTHDC1 promoter was measured. Data present as mean ± SD with three replicates. Ns, not significant; **, *P* < 0.01; ***P < 0.001. **H**, 786-O cells were transfected with indicated siRNAs for 24 h. Then, cells were transfected with EV, GV592-YTHDC1 plasmids WT another 24 h. Cells were harvested and the activity of YTHDC1 promoter was measured. Data present as mean ± SD with three replicates. Ns, not significant; ***P < 0.001. **J **and** K**, 786-O and A498 cells were transfected with indicated siRNAs for 48 h. Cells were collected for western blot analysis (**J**) and RT-qPCR assay (**K**). Data presents as mean ± SD with three replicates. ***, *P* < 0.001. **L** and** M**, 786-O and A498 cells were transfected with empty vector, 1 ng HDAC2 plasmids, or 5 ng HDAC2 plasmids for 24 h. Cells were harvested for western blot analysis (**L**) and RT-qPCR assay (**M**). Data presents as mean ± SD with three replicates. ***, *P* < 0.001. **N**. the ChIP-qPCR was performed by using the IgG or HDAC2 antibodies in 786-O and A498 cells. Data presents as mean ± SD with three replicates. ***, *P* < 0.001. **O**, The ChIP was firstly performed by using the YY1 antibodies. Then, the ChIP-re-ChIP assay was performed by using the IgG or HDAC2 antibodies in 786-O and A498 cells. Data presents as mean ± SD with three replicates. ***, *P* < 0.001. **P**, 786-O and A498 cells were transfected with the indicated siRNAs for 48 h. Cells were collected for western blot analysis

The above results showed that YTHDC1 played an important role in regulating the progression and drug resistance of renal cancer cells, which suggests that YTHDC1 might be an ideal candidate for the treatment of ccRCC. The following experiment was conducted to explore the regulatory mechanism of YTHDC1 in renal cancer cells. The PROMO dataset (http://alggen.lsi.upc.es/cgi-bin/promo_v3/promo/promoinit.cgi?dirDB=TF_8.3) and the GeneCards dataset (https://www.genecards.org/) were applied to predict the potential transcription factors of YTHDC1 (Fig. 7A and B). Among them, YY1 was found in both datasets (Fig. 7A and B). Next, we examined whether YY1 transcriptionally regulated the expression of YTHDC1 in renal cancer cells. We found that knockdown of YY1 increased the protein and mRNA levels of YTHDC1 in 786-O and A498 cells (Fig. 7C and D). We also showed that YY1 inhibition reduced the phosphorylation of ERK in renal cancer cells (Supplementary Fig. 4 A). In addition, the ChIP–qPCR analysis indicated that YY1 was enriched in the promoter region of YTHDC1, and YY1 inhibition decreased this enrichment (Fig. 7E). Moreover, we cloned a DNA sequence containing the potential binding sequence of YY1 and two mutants to generate three GV592-YTHDC1 promoter plasmids (Fig. 7F). We showed that the luciferase activity of the GV592-YTHDC1 WT promoter plasmid was higher than that of the MUT1 or MUT2 plasmid in both 786-O and A498 cells (Fig. 7G). Moreover, we also demonstrated that the luciferase activity of MUT1 was greater than that of MUT2 (Fig. 7G). Furthermore, we showed that knockdown of YY1 decreased the luciferase activity of GV592-YTHDC1 WT in 786-O cells (Fig. 7H). These data suggest that YY1 decreased the expression of YTHDC1 at the transcriptional level. Analysis of the ChIP-atlas dataset also found that histone deacetylase 2 (HDAC2) colocalized with YY1 in the promoter region of YTHDC1 (Fig. 7B). It has been reported that HDAC2 forms a complex with YY1 to regulate downstream target gene expression [37]. Moreover, HDAC2 typically suppresses target gene expression through the deacetylation of histones [38, 39]. Thus, we speculated whether YTHDC1 was repressed by the YY1/HDAC2 complex. Consistent with previous findings, we showed that HDAC2 repression promoted YTHDC1 expression in 786-O and A498 cells (Fig. 7J and K). In contrast, overexpression of HDAC2 decreased the expression of YTHDC1 in renal cancer cells (Fig. 7 L and M). Moreover, ChIP–qPCR and ChIP-re-ChIP analysis demonstrated that the HDAC2/YY1 complex bound to the promoter region of YTHDC1 in 786-O and A498 cells (Fig. 7N and O). We also showed that coknockdown of HDAC2 and YY1 did not further increase the expression of YTHDC1 in renal cancer cells (Fig. 7P).

#### HDAC2 inhibitors enhance the sensitivity of ccRCC to sunitinib

**Fig. 8:**
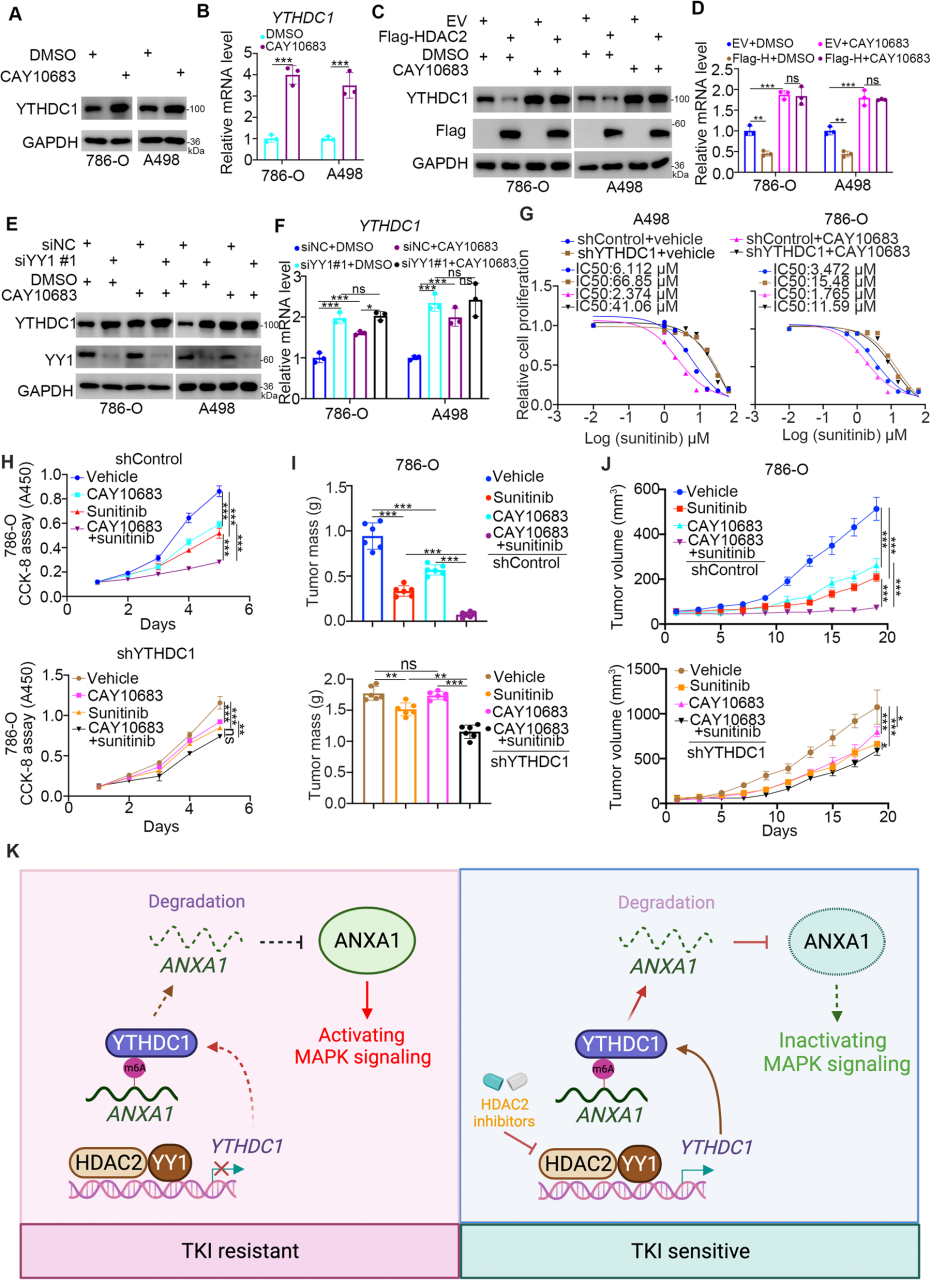
HDAC2 inhibitors enhance the sensitivity of ccRCC to sunitinib. **A **and **B**, 786-O and A498 cells were treated with DMSO or CAY10683 (5 µM) for 24 h. Cells were collected for western blot analysis (**A**) and RT-qPCR assay (**B**). Data presents as mean ± SD with three replicates. ***, *P* < 0.001. **C **and** D**, 786-O and A498 cells were transfected with indicated plasmids for 24 h. Then, these cells were treated with DMSO or CAY10683 (5 µM) for another 24 h. Cells were collected for western blot analysis (**C**) and RT-qPCR assay (**D**). Data presents as mean ± SD with three replicates. Ns, not significant; **, *P* < 0.01; ***, *P* < 0.001. **E **and** F**, 786-O and A498 cells were transfected with indicated siRNAs for 24 h. Then, these cells were treated with DMSO or CAY10683 (5 µM) for another 24 h. Cells were collected for western blot analysis (**C**) and RT-qPCR assay (**D**). Data presents as mean ± SD with three replicates. Ns, not significant; **, *P* < 0.01; ***, *P* < 0.001. **G**, A498 and 786-O cells were transfected with indicated shRNAs for 48 h. Then, these cells were treated with vehicle (DMSO) or CAY10683 (5 µM) for another 24 h. These cells were harvested and treated with a serial dose of sunitinib. These cells were subjected to CCK-8 assay. **H**, 786-O cells were transfected with indicated shRNAs for 48 h. Then, these cells were treated with vehicle (DMSO) or CAY10683 (5 µM) for another 24 h. These cells were subjected to CCK-8 assay. Data presents as mean ± SD with three replicates. Ns, not significant; **, *P* < 0.01; ***, *P* < 0.001. **I** and **J**, 786-O cells were transfected with indicated shRNAs for 72 h. After puromycin selection, cells were collected and subcutaneously injected into the nude mice. The tumor mass was shown in panel I, and tumor growth curve was shown in panel J. Data presents as mean ± SD with six replicates. Ns, not significant; *, *P* < 0.05; **, *P* < 0.01; ***, *P* < 0.001. **K**, a model depicting that the HDAC2/YY1 complex transcriptionally represses the expression of YTHDC1, which prevents the downregulation of ANXA1 induced by YTHDC1 and activates the MAPK pathway to decrease the sensitivity of ccRCC to sunitinib. HDAC2 inhibitors treatment blocks the HDAC2/YY1/YTHDC1/ANXA1/MAPK axis to enhance the anti-tumor effect of sunitinib

To further confirm the regulation of YTHDC1 by the HDAC2/YY1 complex, 786-O and A498 cells were treated with HDAC2-specific inhibitors (CAY10683). We found that CAY10683 treatment increased the protein and mRNA levels of YTHDC1 in renal cancer cells (Fig. 8A and B). We then showed that CAY10683 abrogated the downregulation of YTHDC1 induced by overexpression of HDAC2 (Fig. 8C and D). Moreover, we demonstrated that CAY10683 treatment did not further increase the expression of YTHDC1 in the YY1 knockdown group (Fig. 7E F). In addition, we demonstrated that HDAC2 inhibitor treatment decreased the phosphorylation of ERK in renal cancer cells (Supplementary Fig. 4B). These data suggest that HDAC2 inhibitors also regulate YTHDC1 through the HDAC2/YY1 complex.

Because the downregulation of YTHDC1 contributes to sunitinib resistance in 786-O and A498 cells, we evaluated whether HDAC2 inhibitors overcome sunitinib resistance through YTHDC1. First, we demonstrated that CAY10683 treatment decreased the IC50 values in the shControl group but not in renal cancer cells after YTHDC1 knockdown (Fig. 8G). Next, the CCK-8 assay and nude mouse xenograft assay showed that CAY10683 enhanced the antitumor effect of sunitinib in wild-type renal cancer cells (Fig. 8H-K, Supplementary Fig. 5 A). However, YTHDC1 depletion attenuated the effect of CAY10683 on modulating the sensitivity of sunitinib in vitro and in vivo (Fig. 8H-K, Supplementary Fig. 5 A). Taken together, our data suggest that the HDAC2 inhibitor enhances the antitumor effect of sunitinib through the HDAC2/YY1-YTHDC1 axis in ccRCC (Fig. 8L).

## Discussion

To date, many functions of YTHDC1 have been reported in the literature. For example, YTHDC1 enhances exon inclusion and regulates mRNA splicing by binding with the pre-mRNA splicing factor SRSF3 (SRp20) [23]. Additionally, YTHDC1 is reported to facilitate the nuclear export of mRNAs with m^6^A modification. Mechanistically, YTHDC1 can cooperate with the NXF1 and TREX mRNA export complexes to promote the export of mRNA out of the nucleus [15]. Recently, YTHDC1 has been found to regulate the stability of nuclear mRNAs, including *SQSTM1* and *PTEN* [24, 40]. Similarly, our results demonstrated that YTHDC1 interacts with *ANXA1* mRNA via m^6^A modification and decreases its stability. We also explored the cancer-related role of YTHDC1 in ccRCC. Consistent with previous findings, YTHDC1 is downregulated in ccRCC tissues and functions as a tumor-inhibiting protein. However, as an m^6^A reader, the specific function of YTHDC1 in various types of cancer is controversial. YTHDC1 modulates MCM complex-mediated DNA replication and promotes leukemogenesis [16]. However, YTHDC1 was reported to suppress the progression of glioma by inhibiting VPS25 [41]. Moreover, YTHDC1 was also found to inhibit glycolysis in pancreatic cancer through the miR-30d/RUNX1 axis [42]. The reason YTHDC1 performs different functions in tumors may be related to its differential expression in specific tumors and distinct downstream target genes.

In this study, ANXA1 was proven to be the downstream target gene of YTHDC1 in renal cancer cells. Many studies have mentioned that there is a close relationship between ANXA1 and the chemosensitivity of tyrosine kinase inhibitors [17, 43]. Consequently, we have shown that YTHDC1 regulated the sensitivity of sunitinib through ANXA1/MAPK signaling pathways. It has been well documented that the hyperactivation of MAPK/ERK signaling contributes to TKI resistance in various types of cancer [44, 45]. Inhibition of the MAPK/ERK signaling pathways by specific chemicals overcomes the resistance of TKIs [46]. Here, YTHDC1 acted as a negative regulator of the ERK signaling pathway to sensitize ccRCC to TKI inhibitors. Thus, investigating the regulatory mechanism of YTHDC1 would help identify novel chemicals to enhance the antitumor effect of TKIs in renal cancer cells. Interestingly, we found that the HDAC2/YY1 complex suppressed YTHDC1 expression in renal cancer cells. Furthermore, HDAC2 inhibitors could promote the antitumor effect of sunitinib partially through YTHDC1 in renal cancer cells. Moreover, it has been reported that the ERK signaling pathway modulates the activation of HDAC2 [47]. Our results suggest that HDAC2 may also regulate the activation of ERK, but the relationship between the ERK signaling pathway and HDAC2 needs to be further studied. HDAC2 is an epigenetic regulator that controls global gene expression in cells [48]. Although we showed that HDAC2 inhibitors obtained good results in inhibiting tumor growth in cell and animal experiments, HDAC2 is highly homologous to HDAC8, which might result in unexpected side effects due to the paninhibition of HDAC [49]. Similar to the results of DNMT inhibitors (e.g., 5-aza-dC) in clinical trials [50], HDAC paninhibitors approved by the Food and Drug Administration (FDA) might lead to side effects, including cardiac disturbance (arrhythmias), gastric issues (vomiting), or hematologic imbalance (anemia) [48]. Thus, much attention should be given to the side effects caused by HDAC2 inhibitors in future therapies.

## Conclusion

Our results demonstrated that YTHDC1 is downregulated in ccRCC tissues compared with normal tissues. Low expression of YTHDC1 is associated with a poor prognosis in patients with ccRCC. Subsequently, we showed that YTHDC1 inhibits the progression of renal cancer cells via downregulation of the ANXA1/MAPK pathways. Moreover, we also showed that the YTHDC1/ANXA1 axis modulates the sensitivity of tyrosine kinase inhibitors. We also revealed that HDAC2 inhibitors resensitize ccRCC to tyrosine kinase inhibitors through the YY1/HDAC2 complex. Taken together, we identified a novel YY1/HDAC2/YTHDC1/ANXA1 axis modulating the progression and chemosensitivity of ccRCC.

## Supplementary Information

**Additional file 1.**

## Data Availability

The datasets used and/or analyzed during the current study are available from the corresponding authors on reasonable request.

## Abbreviations

ccRCC: Clear cell renal cell carcinoma
TKIs: Tyrosine kinase inhibitors
TCGA: The Cancer Genome Atlas
CCK-8: Cell counting kit-8
RT‒qPCR: quantitative real time PCR
RIP-qPCR: RNA immunoprecipitation PCR
MeRIP-qPCR: methylated RIP-qPCR
RNA-seq: RNA sequencing
m^6^A: N6-Methyladenosine
AML: acute myeloid leukemia
HCC: hepatocellular carcinoma
ENCORI: Encyclopedia of RNA Interactomes
MAPK: mitogen-activated protein kinase
IACUC: Institutional Animal Care and Use Committee
SDS‒PAGE: sodium dodecyl sulfate‒polyacrylamide gel electrophoresis
IHC: Immunohistochemistry
ChIP: chromatin immunoprecipitation
TNF: tumor necrosis factor
HR: hazard ratio
ERK: extracellular signal-regulated kinases
ANXA1: annexin A1
YTHDC1: YTH domain containing 1
EGFR: epidermal growth factor receptor
