# Supplementary material for: YTHDC1 is downregulated by the YY1/HDAC2 complex and controls the sensitivity of ccRCC to sunitinib by targeting the ANXA1-MAPK pathway
Source: J Exp Clin Cancer Res. 2022 Aug 17;41:250. doi: 10.1186/s13046-022-02460-9 (PMC9382764; doi:10.1186/s13046-022-02460-9)
Supplement: Supplementary file 1 — Additional file 1. [file 13046_2022_2460_MOESM1_ESM.docx]

**YTHDC1 downregulated by the YY1/HDAC2 complex controls the sensitivity of ccRCC to sunitinib through targeting the ANXA1-MAPK pathway**

Wei Li, Kun Ye, Xurui Li, Xinlin Liu, Mou Peng, Fang Chen, Yinhuai Wang, Wei Xiong, Liang Zhu

**
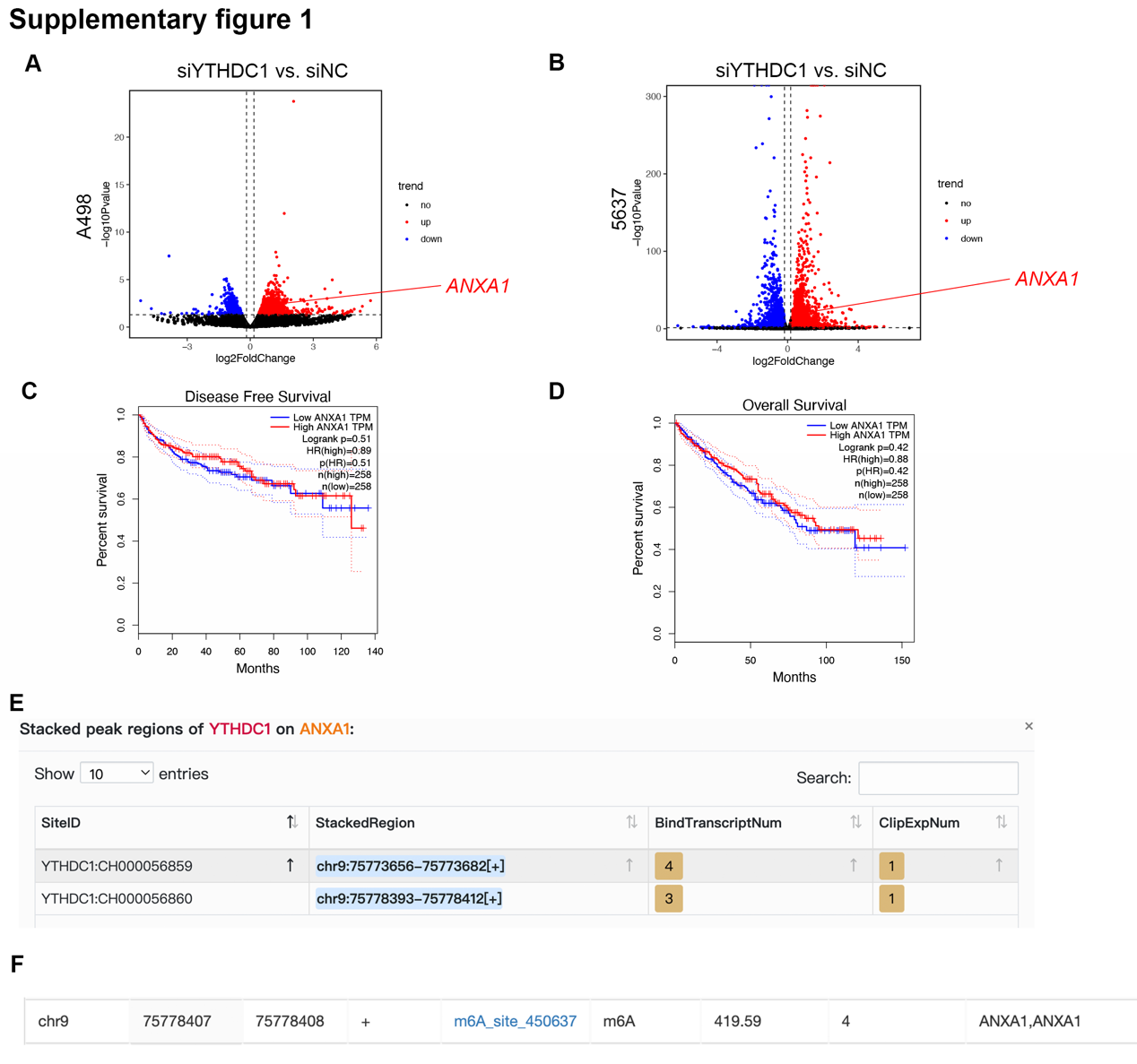
**

**Supplementary figure 1.**

**A,** The RNA-seq of A498 cells after knockdown of YTHDC1**; B,** The RNA-seq of 5637 cells after knockdown of YTHDC1; **C and D**，The GEPIA web tool showed the disease free survival and overall survival of ANXA1 in TCGA-KIRC. P values were indicated in the panel. **E,** ENCORI web tool was used to found the YTHDC1 binding sites on ANXA1; **F**, RMBase v2.0 was used to found the m^6^A modification sites on ANXA1.


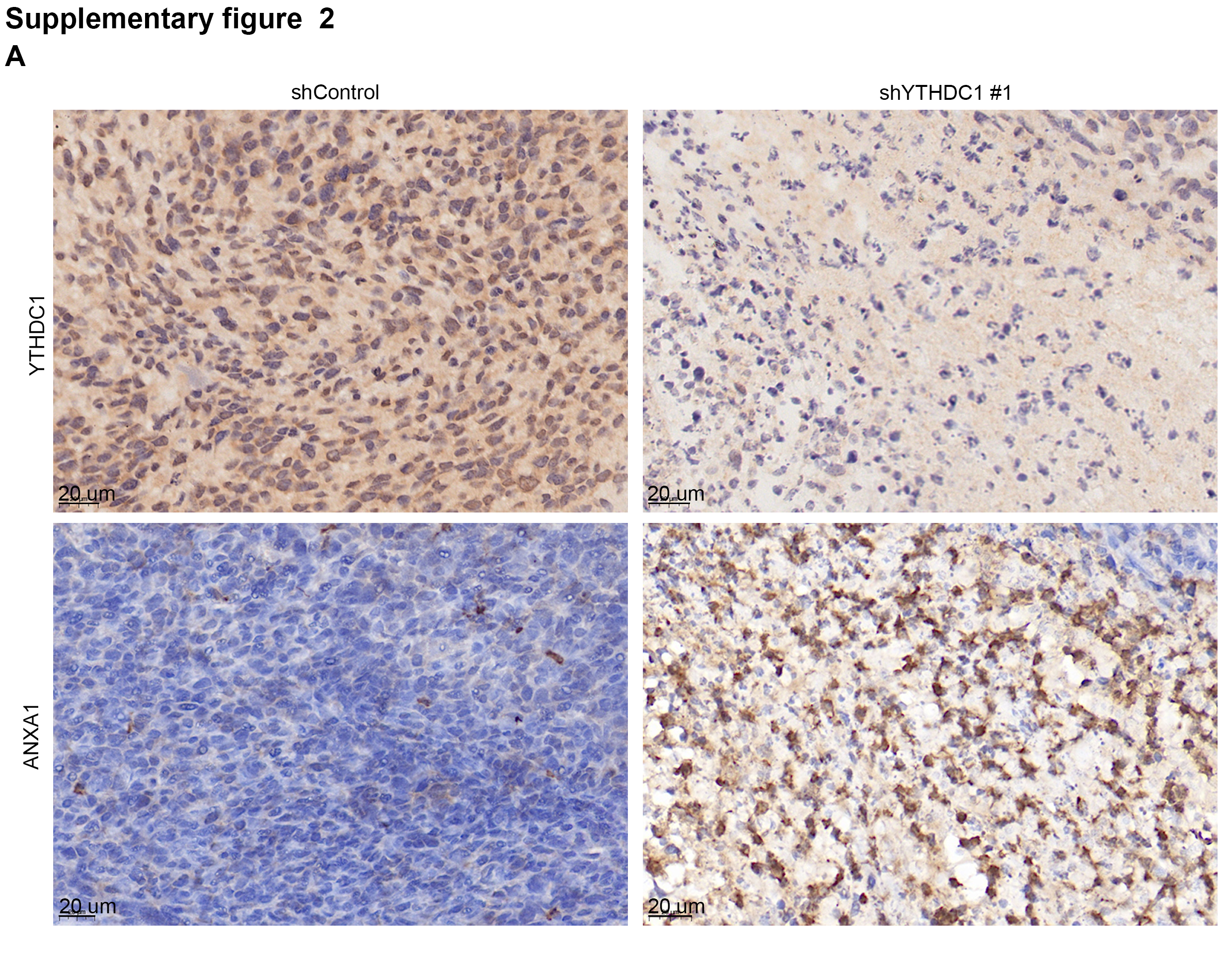


**Supplementary figure 2.**

A, 786-O cells were transfected with indicated shRNAs for 72 h. After puromycin selection, cells were subcutaneously injected into the nude mice. The excised tumors were subjected to IHC staining of YTHDC1 (antibodies dilution 1:1000) or ANXA1 (antibodies dilution 1:1000). The scale bar is 20 μm.


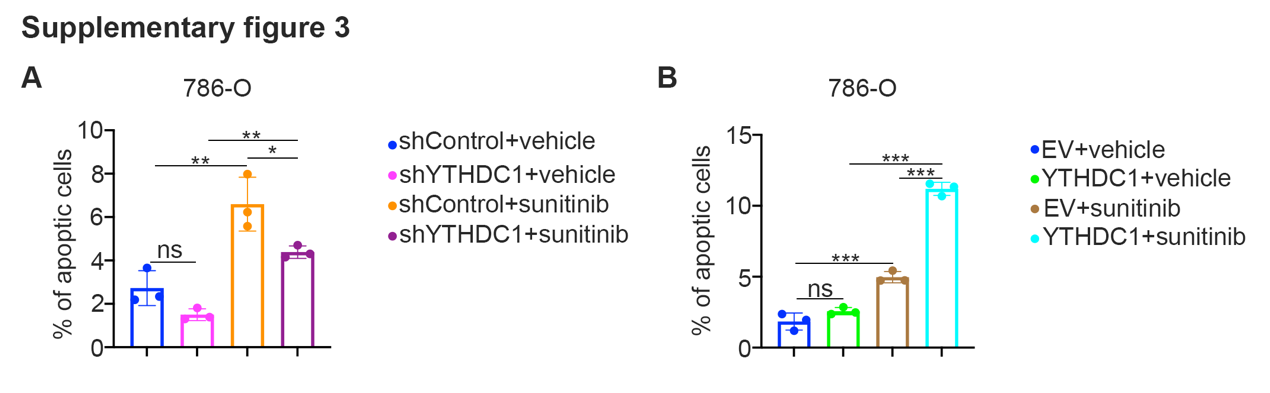


**Supplementary figure 3.**

**A,** 786-O cells were transfected with indicated shRNAs for 72 h. Cells were treated with or without sunitinib (2 μM) and subjected to Annexin V-PI assay. Data presents as mean ± SD with three replicates. Ns, not significant; *, P < 0.05; **, P < 0.01. **B**, 786-O cells were transfected with indicated plasmids for 24 h. Cells were treated with or without sunitinib (2 μM) and subjected to Annexin V-PI assay. Data presents as mean ± SD with three replicates. Ns, not significant; ***, P < 0.001.


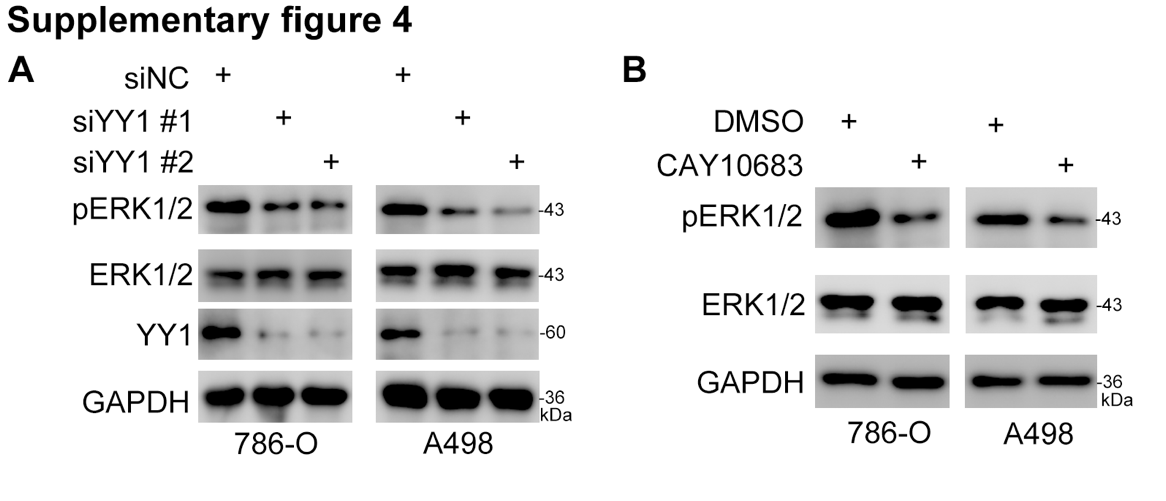


**Supplementary figure 4.**

A, 786-O and A498 cells were transfected with indicated siRNAs for 48 h. Cells were harvested for western blot analysis. **B**, 786-O and A498 cells were treated with indicated DMSO or CAY10683 for 24 h. Cells were harvested for western blot analysis.


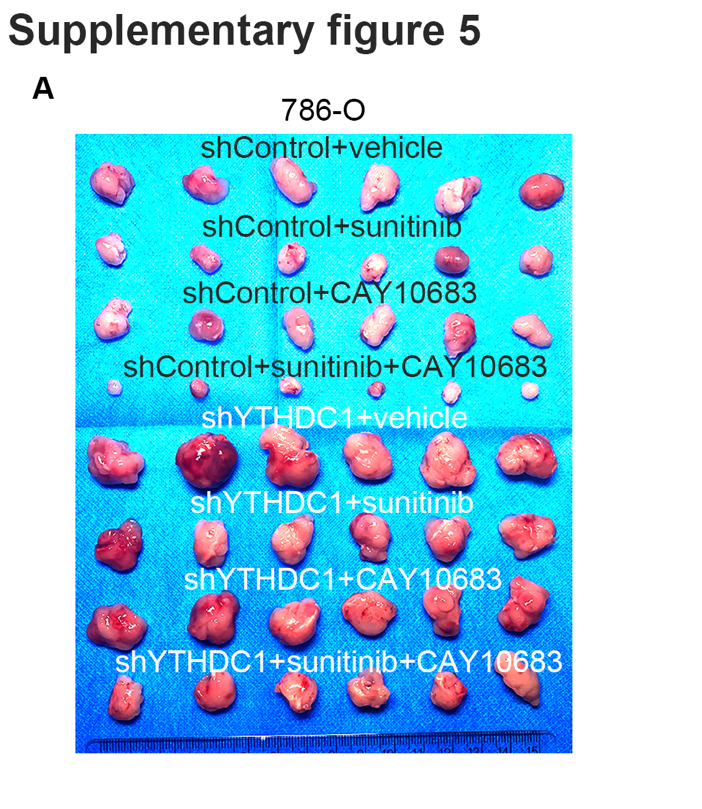


**Supplementary figure 5.**

**A,** 786-O cells were transfected with indicated shRNAs for 72 h. After puromycin selection, cells were collected and subcutaneously injected into the nude mice. The tumor image was shown in panel A.

**Supplementary Material and Methods**

**Cell migration and invasion assay**

For migration assay, cells were transfected with the indicated plasmids and cultured to confluence on 6-well plates. The cell layer was scratched and detached cells were removed. For each sample, at least three scratched fields were photographed immediately. Cell migration was evaluated by measuring the cell-covered area.

The in vitro cell invasion assay was performed using a BioCoat Matrigel invasion chamber (BD Biosciences) according to the protocol of the manufacturer. Cells were cultured in the insert for 24 h. Cells were fixed in methanol for 15 min and then stained with 1 mg/ml crystal violet for 20 min. At least five fields for each group were photographed after staining, and invaded cells were counted.

**Table S1. The siRNA and shRNA sequences.**

| siYY #1 | 5'- GTGGTTGAAGAACAGATCATTGG-3' |
| --- | --- |
| siYY #2 | 5'- AACCTGAAATCTCACATCTTAAC-3' |
| siHDAC2 #1 | 5'- CGGTGATATTGGAAATTATTATT-3' |
| siHDAC2 #2 | 5'- GTCAATAAGACCAGATAACATGT-3' |
| shANXA1 #1 | 5′- CCGGGCATTCTATCAGAAGATGTATCTCGAGATACATCTTCTGATAGAATGCTTTTTG -3′ |
| shANXA1 #2 | 5′- CCGGGCCTTGTATGAAGCAGGAGAACTCGAGTTCTCCTGCTTCATACAAGGCTTTTTG -3′ |
| shYTHDC1 #1 | 5'-CACCAGGAGAAAGATGGAGAACTTAATCTCGAGATTAAGTTCTCCATCTTTCTCC-3' |
| shYTHDC1 #2 | 5'-CACCATCGGAAAATTCGTCTATCAAGTCTCGAGACTTGATAGACGAATTTTCCGA-3' |

**Table S2. The primer sequences for RT-qPCR.**

| Gene（Human） | Forward primer (5′ - 3′) | Reverse primer (5′ - 3′) |
| --- | --- | --- |
| actin beta | ACAGAGCCTCGCCTTTGCC | TGGCCATCTCTTGCTCGAAG |
| YTHDC1 | GGAGGGCCAAATCTCCTACG | CTTTTCGGACAGCACGAACG |
| ANXA1 | ACTGCTTCTACAGGATTTATGGTT | CAAAAAGCAGCCCCCATCAC |

**Table S3. The primer for ChIP-qPCR**

| Gene（Human） | Forward primer (5′ - 3′) | Reverse primer (5′ - 3′) |
| --- | --- | --- |
| YTHDC1（YY1/HDAC2） | AGAAAATTATTCGGCACTGTGT | TCCTGCCTCTGTCTCCCTAA |

**Table S4. The primer for RIP-PCR**

| Gene（Human） | Forward primer (5′ - 3′) | Reverse primer (5′ - 3′) |
| --- | --- | --- |
| ANXA1（YTHDC1） | GAGGTTTGGAGGGGAAGAAC | CGGTCACCCTGAAAGAAATC |

**Table S5. The primer for MeRIP-qPCR**

| Gene（Human） | Forward primer (5′ - 3′) | Reverse primer (5′ - 3′) |
| --- | --- | --- |
| ANXA1 | GAGGTTTGGAGGGGAAGAAC | CGGTCACCCTGAAAGAAATC |
